# Digital pharmacoepidemiology of angiotensin-converting enzyme inhibitors in Russia

**DOI:** 10.3389/fphar.2026.1881727

**Published:** 2026-07-23

**Authors:** Stanislav Kotlyarov, Anna Kotlyarova

**Affiliations:** 1 Department of Nursing, Ryazan State Medical University, Ryazan, Russia; 2 Department of Pharmacy Management and Economics, Ryazan State Medical University, Ryazan, Russia

**Keywords:** ACE inhibitors, digital epidemiology, hypertension, infodemiology, search queries, self-medication, treatment adherence, yandex

## Abstract

**Introduction:**

Hypertension remains one of the leading causes of cardiovascular morbidity and mortality; however, patient adherence to treatment remains inadequate. In the context of a digital society, search queries can serve as an additional source of information regarding the population’s information needs and patterns of interaction with medications.

**Objectives:**

To conduct a comprehensive longitudinal infodemiological study of the Russian population’s interest in angiotensin-converting enzyme (ACE) inhibitors based on an analysis of search queries for the period 2018–2025.

**Methods:**

A retrospective study was conducted using aggregated data from the Yandex search engine (Wordstat) covering 84 months. Search queries were analyzed for six major ACE inhibitors (captopril, enalapril, lisinopril, perindopril, ramipril, fosinopril), as well as for their brand names (21 drugs in total). We analyzed time series (Mann–Kendall test), seasonality (Kruskal–Wallis test), and variability, as well as performed multidimensional semantic analysis (Latent Dirichlet Allocation (LDA), Term Frequency–Inverse Document Frequency (TF-IDF) + k-means, k-medoids, n-grams).

**Results:**

The total volume of analyzed queries exceeded 102 million. The greatest informational interest was identified for the captopril (30.1% of all queries), perindopril (27.9%), and enalapril (21.3%) groups. Perindopril demonstrated the strongest positive trend (τ = 0.762; p < 0.001). Statistically significant seasonality (p < 0.05) was observed for all six main International Nonproprietary Names (INN), with a peak during the winter months and a decline in activity during the summer. Semantic analysis revealed distinct differentiation in information patterns: captopril was associated with emergency use and sublingual administration, enalapril with manufacturer selection, lisinopril with searches for instructions and reviews, and perindopril with fixed-dose combinations. The proportion of queries related to emergency use was highest for captopril (0.53%) and lowest for the other drugs.

**Conclusion:**

Users’ search activity reflects consistent differences in the perception of ACE inhibitors and demonstrates pronounced seasonal dynamics. The identified digital patterns may indirectly reflect medication-related information needs and potential gaps in the population’s health literacy. The infodemiological approach represents a promising tool for monitoring patients’ information needs and optimizing clinical communication and preventive programs.

## 1 Introduction

Hypertension is a significant medical and social problem, as it is highly prevalent among the adult population, underdiagnosed, and often not treated effectively (Kario et al., 2024; Goorani et al., 2025; Inoue, 2025). Crucially, many patients do not consider elevated blood pressure readings to be a problem or do not take an interest in them; consequently, they do not adhere to regular therapy and thus fail to achieve target blood pressure levels (Peacock and Krousel-Wood, 2017; Ashoorkhani et al., 2018). At the same time, hypertension is one of the key risk factors for serious cardiovascular diseases such as myocardial infarction and stroke (Poznyak et al., 2022; Anyaogu et al., 2025; Writing Committee Members et al., 2025; Brown et al., 2026).

The current arsenal of medications allows for the control of hypertension in most cases (Guerrero-García and Rubio-Guerra, 2018; McCarthy et al., 2025; Writing Committee Members et al., 2025). An important prerequisite is the selection of an appropriate medication and dosage, as well as the patient’s adherence to the physician’s recommendations. Angiotensin-converting enzyme (ACE) inhibitors have remained one of the cornerstones of pharmacotherapy for cardiovascular diseases for decades (Khalil et al., 2001; Guerrero-García and Rubio-Guerra, 2018; Singh et al., 2025). Thanks to their proven cardioprotective and nephroprotective properties, they are included in key guidelines for the treatment of hypertension, chronic heart failure, ischemic heart disease, and diabetic nephropathy (Alcocer et al., 2023; Herman et al., 2023; McEvoy et al., 2024; Singh et al., 2025; Writing Committee Members et al., 2025). In this regard, ACE inhibitors are widely used medications that are actively employed in real-world clinical practice (Turner and Kodali, 2020; Rouette et al., 2022; Alcocer et al., 2023; Herman et al., 2023; Singh et al., 2025). Despite their relative safety and ease of use, the problem of poor patient adherence to treatment persists, and accurately assessing this adherence is difficult (Abegaz et al., 2017; Peacock and Krousel-Wood, 2017; Ashoorkhani et al., 2018; Wawruch et al., 2023; Arshed et al., 2025).

Traditional pharmacoepidemiology, based on data from prescriptions, sales, and registries, provides important information on drug use at the population level (Garbe and Suissa, 2014; Rouette et al., 2022; Beyene et al., 2023; Sabaté and Montané, 2023). However, it has certain limitations, often failing to capture early behavioral trends, hidden interest in self-medication, patient doubts, or searches for information on side effects. In the era of widespread digitalization, the public increasingly turns to internet searches as the first, and sometimes the primary, source of health information, including information on medications. This digital footprint, in the form of thousands of search queries, forms a new, rich paradigm for public health research known as infodemiology or digital epidemiology. In this context, it is interesting to examine how often people search for various ACE inhibitors, as well as what information about these medications they are seeking. These data are important not only for assessing pharmacoepidemiology but also have clinical relevance, as they allow us to evaluate the public’s information needs and the questions, they have not asked their treating physicians.

The novelty of this study lies in the application of digital epidemiology methods to analyze the public’s information needs. Unlike traditional surveys and focus groups, the analysis of search queries provides objective data on the public’s spontaneous information needs, free from socially desirable responses. The practical significance of this work lies in the potential to apply the findings to solve practical problems, ranging from optimizing pharmacy product assortment policies or improving the effectiveness of pharmaceutical marketing to addressing clinical challenges, such as developing targeted educational programs for patients or enhancing government drug provision programs.

Thus, the aim of this study was to conduct a comprehensive, longitudinal study of the digital epidemiology of the Russian population’s interest in ACE inhibitors through a retrospective analysis of search query data over a 7-year period (2018–2025).

## 2 Materials and methods

### 2.1 Study design and data sources

A retrospective infodemiological (digital epidemiology) study was conducted based on an analysis of large aggregated data from the Yandex search engine. The public analytical tool Yandex. Wordstat (Author Anonymous, 2026) was used as the source, providing statistics on the frequency of search queries for specified keywords. The analysis covered the period from November 1, 2018, to October 30, 2025 (inclusive), totaling 84 full months. This period was chosen due to the need to assess long-term trends and cover several seasonal cycles in the population’s information behavior.

### 2.2 Selection of drugs and formation of the semantic search core

For the analysis, a complete list of ACE inhibitors registered and available on the pharmaceutical market of the Russian Federation during the study period was selected. The sample was formed based on official data from the State Register of Medicines, as well as information from the RLSNET® information and analytical system (“Russian Register of Medicines”) (Russian Register of Medicines, 2026). The selection of specific trade names (brands) was carried out using the Vyshkovsky Index—an indicator unique to the Russian pharmaceutical market that reflects the level of information demand for a drug among the professional community (medical and pharmaceutical specialists) (Vyshkovsky index, 2026). The index is calculated as the ratio of the number of queries for a specific brand to the total number of queries for all brands in the RLSNET® system over a given period and is expressed in per mille (‰).

The following principles were applied when forming the final sample:

All original medicinal products present on the market during the study period were included in the analysis regardless of the quantitative value of the Vyshkovsky index, as they are reference products and are of fundamental importance for assessing consumption patterns and pharmacoeconomic indicators. The exception was the original ramipril drug (“Tritace”), which was not supplied to the Russian Federation during the specified period.

Selection of generic drugs. Generic drugs were included based on a threshold value of the Vyshkovsky index >0.02‰. This allowed for the exclusion of drugs with extremely low or sporadic information demand, which in fact have no significant impact on consumption patterns and pharmacoepidemiological indicators on a national scale.

Prevention of double counting. The analysis revealed that for a number of active ingredients (e.g., captopril), the generic is marketed under a trade name that coincides with the International Nonproprietary Name (INN) in Russian. Since, in addition to queries for specific brands, aggregated queries directly for INNs (e.g., “captopril,” “enalapril,” “lisinopril”) were taken into account, separate accounting for such generics would lead to an artifactual duplication of the information contribution of the same active ingredient. To avoid this methodological artifact, generics whose trade names are identical to the INN were not included in the list of analyzed brands; their information demand is considered fully accounted for within the corresponding indicator for the INN.

A complete list of INNs and brand names selected according to the criteria described, along with their category (brand-name drug/generic), is presented in Table 1.

**TABLE 1:** List of ACE inhibitors included in the analysis, with their category and key brand names.

INN	Vyshkovsky index, ‰	Category	Trade names (brands)	Vyshkovsky index, ‰
Captopril	1.525	Original	Capoten	2.191
		Generic	— (Same name as INN)	—
Enalapril	1.655	Original	Renitec	0.028
		Generic	Enap	0.559
			Enam	0.184
			Berlipril	0.025
			Renipril	0.030
Lisinopril	1.378	Original	—	—
		Generic	Diroton	0.679
Perindopril	2.011	Original	Prestarium	0.364
		Generics	Perineva	0.661
			Parnavel	0.020
Ramipril	0.440	Original	Tritace (not supplied in Russia at the time of the study, therefore not included in this study)	—
		Generics	Hartil	0.117
			Amprilan	0.047
Fosinopril	0.111	Original	Monopril	0.020
		Generics	Fosinap	0.021
			Fozicard	0.056

Thus, the analysis included six INNs and 15 brand names (both originator drugs and generics), for a total of 21 items analyzed.

### 2.3 Data collection and preliminary processing procedure

For each drug, using the Yandex. Wordstat functionality, the number of monthly search queries for the entire study period was extracted (i.e., the number of queries estimated by Yandex corresponding to the entered word or phrase). The search was performed by entering the drug name (international nonproprietary name or brand name) as is, without using any additional operators or filters. All word forms corresponding to the entered name (international nonproprietary name or trademark) were taken into account. Queries unrelated to the drug (place names, proper nouns, homonyms) were excluded from the subsequent semantic analysis. The relative share of queries (%) out of the total number of search queries on Yandex for the corresponding month was used as a normalization factor, which allowed us to account for the overall growth in internet activity and ensure the comparability of data over time.

### 2.4 Semantic and content analysis

To ensure a qualitative interpretation of the data, a content analysis was conducted on the complete list of relevant queries provided by Yandex. Wordstat. The goal of the analysis was to identify keywords, contextual clusters, and unique semantic patterns that shape the digital image of each drug.

To minimize false positives, queries that could be interpreted in multiple ways or were not directly related to the medication were excluded from the semantic core. Semantic analysis was conducted using search query data from November 2025 (period: October 30, 2025 – November 30, 2025). This month was chosen as it marks the onset of seasonal growth in search activity and provides sufficient statistical power for semantic analysis.

The following complementary methods were applied for a comprehensive analysis of the search query structure:

#### 2.4.1 Latent Dirichlet Allocation (LDA) thematic modeling

Latent Dirichlet Allocation was used to identify hidden thematic structures in the corpus of search queries (Kim and Kim, 2022). The analysis was conducted using the topicmodels package in the R environment (v.4.3.2). Based on an analysis of sensitivity (k = 2–8) and thematic coherence (UMass measure), as well as the clinical interpretability of the data for each drug, a five-theme model (k = 5) was constructed. The top 10 words for each theme were extracted based on beta probabilities (β), reflecting the probability of a word belonging to a given theme.

#### 2.4.2 K-means clustering based on Term Frequency–Inverse Document Frequency (TF-IDF)

To refine the semantic patterns, K-means clustering of TF-IDF vectors was performed. The analysis was conducted using the tidytext and factoextra packages. Based on the maximization of the silhouette coefficient in the range k = 5–10 and the clinical interpretability of the data for each drug, eight clusters were identified (k = 8).

#### 2.4.3 K-medoids clustering based on the Levenshtein distance

To evaluate clustering based on the character similarity of queries, the k-medoids (PAM) method was applied using a Levenshtein distance matrix. The analysis was performed using the stringdist and cluster packages. Based on sensitivity analysis (k = 3–8) and the maximum silhouette coefficient, as well as the clinical interpretability of the data for each drug, six clusters (k = 6) were identified based on the top 500 most frequent queries.

#### 2.4.4 N-gram analysis (bigrams and trigrams) with thematic clustering

To identify stable word combinations and phrase patterns, an n-gram analysis was conducted, identifying bigrams (2-g) and trigrams (3-g) (Senti-N-Gram: An n-gram lexicon for sentiment analysis, 2018; Performance Study of N-grams in the Analysis of Sentiments (2025). The analysis was performed using the tidytext and dplyr packages. The procedure included the following steps:

Extraction of n-grams: For each drug, bigrams and trigrams were extracted from the search queries, taking into account the frequency of occurrences. The minimum frequency threshold for inclusion in the analysis was 30 for bigrams and 20 for trigrams.

Thematic n-gram clustering: All identified n-grams were automatically assigned to 13 thematic clusters based on the presence of keywords using regular expressions. The clusters included: Instructions (instructions, use, method, indications, dosage); Dosage (how much, dose, mg, tablets, take, drink); Reviews/Opinions (reviews, opinion, doctors, patients, helps, effect); Price/Commercial (price, buy, cost, pharmacy, rubles); Fixed combinations (indapamide, amlodipine, combination, plus); Manufacturers (Teva, Hexal, Reneval, Vertex, Velfarm, Canon, Ozon, Akos, FPO, Stada, Alium); Sublingual (under the tongue, dissolve, sublingually, under the tongue); Emergency (ambulance, aid, crisis, urgent, emergency, immediate); Comparison (which is better, or, difference, comparison, instead of, substitute, equivalent); Safety (side effects, contraindications, harm, danger, overdose, poisoning, allergy); Simple explanations (in simple terms, what it helps with); Time of action (how long after, duration of action, fast, time); Regular therapy (every day, constantly, daily, course).

Data aggregation by clusters: For each thematic cluster, the total frequency of mentions was calculated by summing the frequencies of all n-grams assigned to that cluster. It is these aggregated values (rather than the frequencies of individual n-grams) that were used for a quantitative comparison of semantic patterns between drugs. This approach allows us to account for all possible variations of queries related to a single topic and avoid an artifactual underestimation of the topic’s relevance due to the lexical diversity of user queries.

Analysis of individual n-grams: Along with aggregated data by clusters, the text provides the frequencies of the most representative bigrams and trigrams to illustrate specific query formulations. For perindopril, an additional analysis was conducted of trigrams specifically associated with fixed combinations (containing “indapamide,” “amlodipine,” “plus”).

#### 2.4.5 Targeted analysis of emergency queries

To identify queries related to the emergency use of medications, a targeted analysis of keywords was conducted using regular expressions. Keywords included: “ambulance,” “crisis,” “hypertensive crisis,” “emergency,” “urgent,” “emergency care”. Queries were classified into the following patterns: Ambulance, Crisis, Urgent.

#### 2.4.6 Quantitative analysis of keywords

To determine the proportion of queries related to specific thematic categories, a quantitative analysis of keywords was conducted using regular expressions, following the same principle as n-gram clustering. The categories included: “instructions,” “dosage,” “reviews,” “emergency care,” “fixed combinations,” “manufacturers,” and “sublingual administration.” For each drug, the percentage of queries containing the corresponding keywords was calculated out of the total number of queries.

When presenting the results of the n-gram analysis in the text and Table 2, aggregated frequencies by thematic clusters are used (for example, the “Manufacturers” cluster includes all bigrams containing manufacturers’ names). In cases where the text provides frequencies of individual n-grams (e.g., “lisinopril teva”), these values are part of the corresponding cluster and reflect the contribution of a specific bigram to the cluster’s total frequency. This approach provides both a macroscopic (at the cluster level) and a microscopic (at the level of individual phrases) characterization of the semantic structure of search queries.

**TABLE 2:** Key semantic characteristics of the drugs (% of total mentions).

Feature	Captopril	Enalapril	Lisinopril	Perindopril
Instructions	27.34	33.96	37.34	33.81
Dosage	21.14	19.96	20.87	22.86
Reviews	12.06	17.29	19.72	18.22
Emergency use	0.53	0.04	0.00	0.00
Fixed-dose combinations	<0.05	0.80	1.76	10.81
Manufacturers	2.78	6.74	3.12	4.77
Sublingual administration	3.13	0.11	0.07	0.01
Additional data from the bigram analysis (absolute frequency of occurrences)				
Bigram cluster	Captopril	Enalapril	Lisinopril	Perindopril
Fixed-dose combinations	466	5,624	13,638	155,698
Manufacturers	33,820	66,355	23,704	94,720
Sublingual administration	35,584	1,001	513	233
Emergency care	6,839	339	0	0
Comparison	42,117	27,797	47,363	82,808

#### 2.4.7 Selection of the number of clusters

The number of clusters is fixed for all drugs to ensure the comparability of semantic profiles. The choice of k = 8 (k-means), k = 6 (k-medoids), and k = 5 (LDA) is justified by sensitivity analysis, formal metrics (silhouette coefficient, UMass coherence), and expert assessment of the interpretability of the results. Automatic optimization of the number of clusters (e.g., based on the maximum silhouette coefficient) led to the loss of clinically significant but statistically rare patterns (in particular, for captopril—the “Emergency Care” cluster, accounting for only 0.53% of queries). Therefore, the choice of k was based on a balance between formal quality metrics and the clinical interpretability of the results.

### 2.5 Statistical analysis

Statistical data analysis was performed using the R software environment (v. 4.3.2). Descriptive statistics are presented as the arithmetic mean (M) and standard deviation (SD) to characterize the central tendency and dispersion of the data, as well as the median and interquartile range (Me [Q1; Q3]) for continuous data. To assess the stability of information interest, the coefficient of variation (CV = SD/M × 100%) was calculated.

Time series trend analysis was performed using the nonparametric Mann-Kendall test. To quantitatively assess the direction and strength of the trend, the Kendall correlation coefficient (τ) was calculated, taking values from −1 (strictly decreasing trend) to +1 (strictly increasing trend). The statistical significance of the trend was determined based on the p-value of the Mann–Kendall test. The value of τ was interpreted as follows: |τ| < 0.3 — weak, 0.3–0.5 — moderate, >0.5 — strong.

The presence of statistically significant seasonal fluctuations in the monthly time series was tested using the nonparametric Kruskal–Wallis test. For pairwise comparisons between months, Dunn’s *post hoc* test was applied with Holm’s correction for multiple comparisons. The strength of the seasonal effect was estimated by calculating the relative seasonal decline (in %) [(Peak value–Minimum value)/Peak value] * 100%. In addition, pairwise Wilcoxon rank-sum tests (Mann–Whitney U tests) with Bonferroni correction for multiple comparisons was used to compare the four seasons (winter, spring, summer, and fall) in pairs.

To determine seasonality, the effect size was estimated using the epsilon-squared (ε^2^) statistic. To exclude long-term trends prior to the seasonality analysis, STL decomposition (Seasonal-Trend decomposition using Loess) with a periodic seasonal window was applied. For a visual comparison of the shapes of seasonal profiles across drugs with different query volumes, a z-transformation was applied (standardization for each drug separately: z = (x − mean)/SD over 12 months; units are standard deviations), and the results are presented as a heat map. Additionally, to verify the specificity of seasonality relative to overall internet activity, the seasonal analysis was repeated on a normalized series: the proportion of queries out of the total number of all queries in Yandex for the month. For multiple comparisons of trends between drugs, the Benjamini–Hochberg correction for the false discovery rate (FDR) was applied. The resistance of trends to temporal autocorrelation in the series was tested using the modified Mann–Kendall test with the Hamed–Rao correction (Hamed and Ramachandra Rao, 1998), which involves correcting the variance of the test statistic by the autocorrelation coefficient in the series.

All statistical analyses were performed in R version 4.3.2 (Author Anonymous, 2025) (R Core Team, 2025) using the following packages: tidytext (Silge and Robinson, 2016; Queiroz et al., 2025), factoextra (Kassambara et al., 2026), stringdist (van der Loo, 2024), cluster (Maechler et al., 2026), boot (Canty et al., 2025), rcompanion (Silge and Robinson, 2016), dunn. test (Dinno, 2026), and Kendall (McLeod, 2025), topicmodels (Grün et al., 2024), e1071 (Meyer et al., 2025) and modifiedmk (Patakamuri and O’Brien, 2021).

### 2.6 Ethical considerations

The study was conducted using exclusively aggregated, publicly available, and fully anonymized data that does not allow for the identification of individual users. In accordance with the Declaration of Helsinki and national ethical standards, such a study does not require approval from a local ethics committee or informed consent from participants.

## 3 Results

### 3.1 Frequency of searches for ACE inhibitors

**FIGURE 1:**
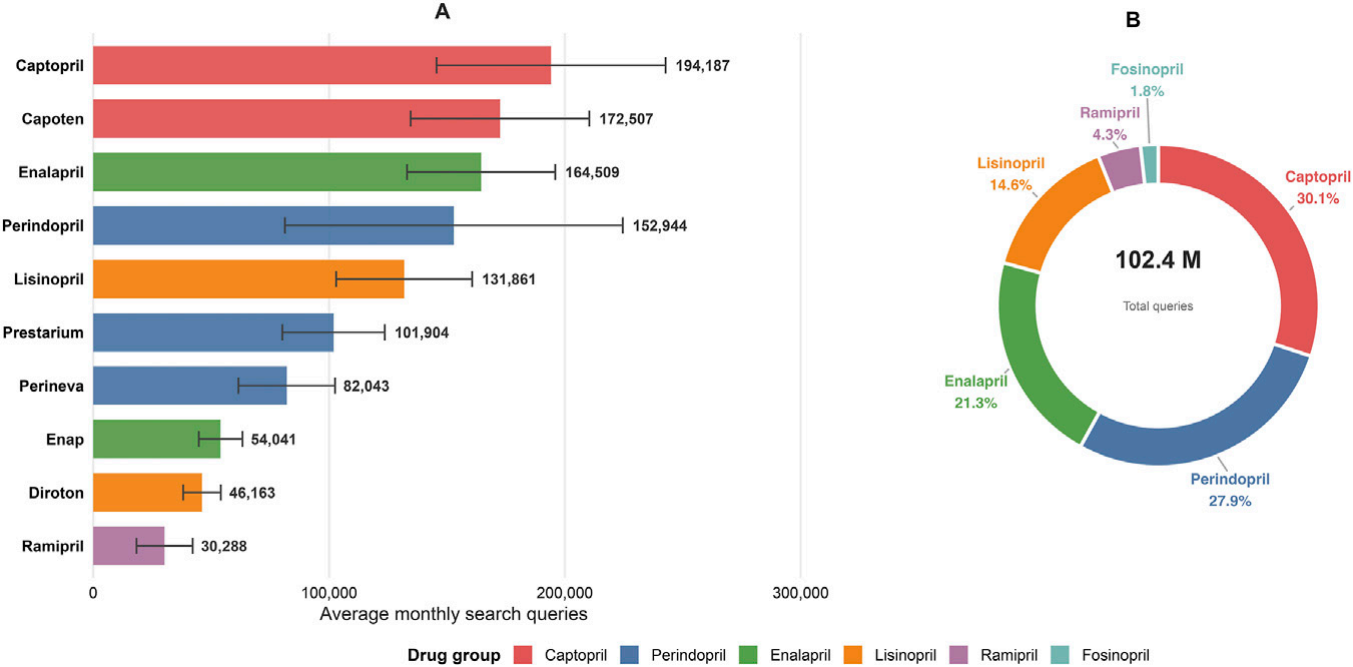
Breakdown of search queries for ACE inhibitors. **(A)** Top 10 ACE inhibitors, ranked by average monthly search volume for the period from November 2018 to October 2025. Captopril ranks first with an average of 194,187 monthly queries, followed by Capoten (172,507), enalapril (164,509), perindopril (152,944), and lisinopril (131,861). Bars represent the mean monthly number of search queries for each drug, and error bars represent the mean ± standard deviation (SD) of the monthly query counts across the 84-month study period (November 2018 – October 2025). **(B)** Distribution of search queries by drug group. The captopril group (including captopril and Capoten) accounts for 30.1% of all queries related to ACE inhibitors, followed by the perindopril group (27.9%) and the enalapril group (21.3%). The total number of queries analyzed was approximately 102.4 million.

Over the analyzed period (84 months), the total volume of search queries showed marked heterogeneity among drugs. The analysis results demonstrated the dominance of captopril in the information field. Throughout the entire study period, the total search traffic for captopril (including searches by the INN and the key brand name “Capoten”) consistently accounted for the absolute maximum (Figure 1A)). The average monthly number of queries for captopril was 194,187 (median—192,148), and for Capoten—172,507 (median—171,256), which together constitute the largest segment of information demand among all ACE inhibitors. The share of captopril-based drugs in the total volume of queries related to this pharmacological group was 30.1% (Figure 1B). This is explained by the wide availability of captopril, its use for both routine therapy and the relief of hypertensive crises, as well as its long history on the Russian pharmaceutical market.

Enalapril also demonstrated high information demand: the average monthly volume of queries by INN was 164,509 (median—162,015), which is the second-highest figure among all INNs and ACE inhibitors after captopril (Figure 1A). Taking into account all brand names included in the analysis (“Renitec,” “Enap,” “Enam,” “Berlipril,” “Renipril”), the total volume for the enalapril group reaches 259,518 queries per month, which corresponds to 21.3% of the total information flow and is surpassed only by the captopril (30.1%) and perindopril (27.9%) groups (Figure 1B).

**FIGURE 2:**
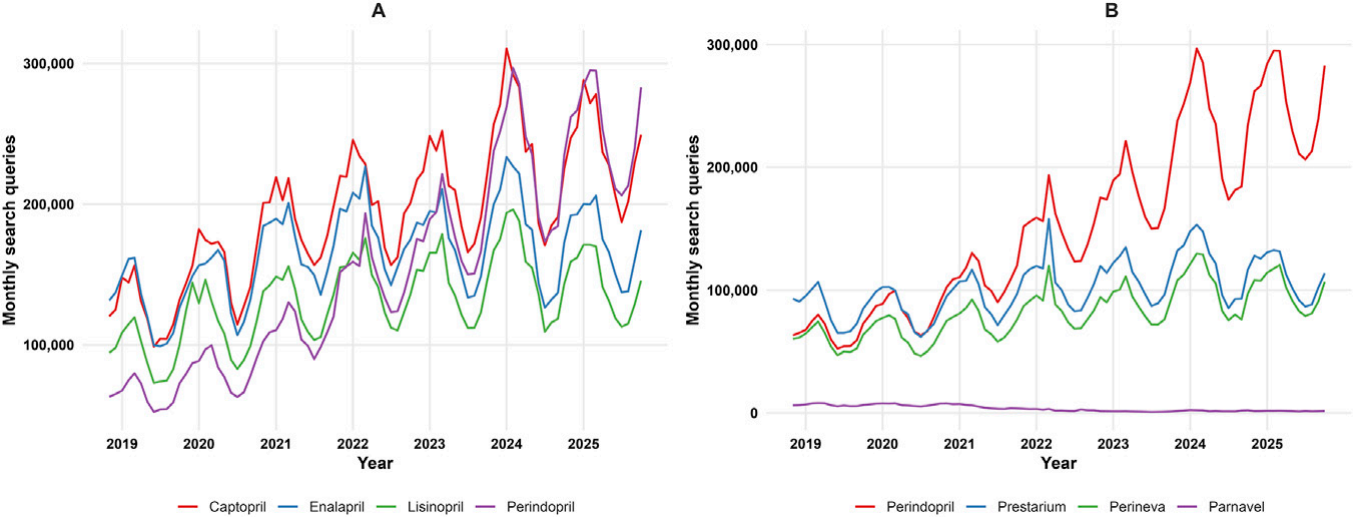
Infodemiological analysis of ACE inhibitors in Russia. **(A)** Trends in search queries for major ACE inhibitors. Line graphs showing the monthly number of queries for captopril, enalapril, lisinopril, and perindopril for the period from November 2018 to October 2025. Captopril consistently holds the leading position, which is explained by its widespread use for both routine therapy and the relief of hypertensive crises. A clear seasonal trend is evident, with peak activity during the winter months. **(B)** Search trends for “perindopril” (brand-name drug and generics). Comparison of search activity for the brand-name drug “Prestarium,” the generics “Perineva” and “Parnavel,” and the INN “perindopril”. Since 2020–2021, search queries for the generic “Perineva” have consistently exceeded those for the original “Prestarium,” reflecting a trend toward import substitution and growing interest in Russian equivalents.

Lisinopril (INN) has an average monthly search volume of 131,861 queries (median: 131,210) (Figure 2A), and when including the brand “Diroton,” the group’s total monthly search volume reaches 178,024 queries (14.6%). The consistently high interest in lisinopril is consistent with its positioning in clinical guidelines as a first-line drug, which is due to its pharmacoeconomic affordability, inclusion in drug benefit lists (DBLs), and the convenience of once-daily dosing, which improves compliance.

The analysis results showed consistently high interest in perindopril with the highest growth rate. Perindopril (brand names “Prestarium,” “Perineva,” “Parnavel”) demonstrated the fourth-highest level of search demand, with a monthly average of 152,944 queries (median: 150,413) (Figure 2B). This high and sustained interest is consistent with perindopril’s positioning in clinical guidelines as a drug with additional vasoprotective and vasoremodeling properties, as well as its widespread use in fixed-dose combinations (e.g., with amlodipine).

The analysis revealed that search queries for ramipril and fosinopril were niche in nature. The volume of search queries for ramipril (“Hartil,” “Amprilan”) and fosinopril (“Monopril,” “Fozicard,” “Fozinap”) remained at a significantly lower level throughout the entire period: a monthly average of 30,288 and 7,431, respectively, and their combined share of the total information flow amounted to only 6.1% (1.3% for fosinopril and 4.3% for ramipril).

### 3.2 Temporal trends in search interest

**FIGURE 3:**
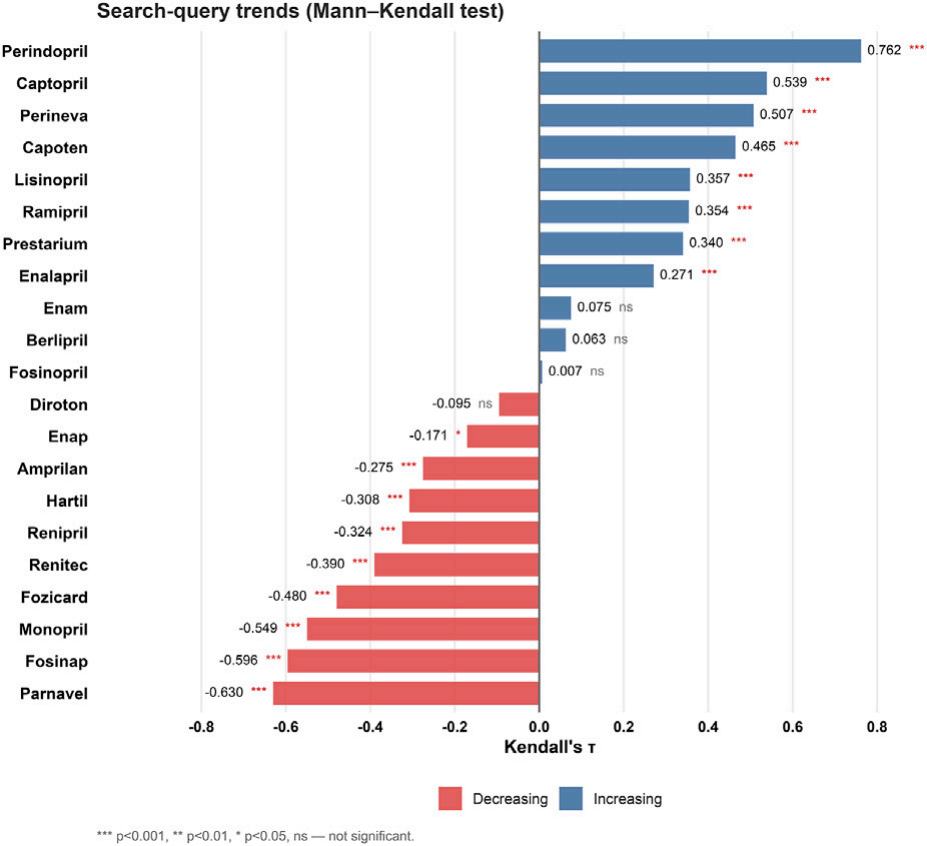
Analysis of search query trends for ACE inhibitors (Mann-Kendall test). The graph shows Kendall’s correlation coefficient (τ) values with significance levels indicated. Positive values of τ correspond to an increasing trend, while negative values correspond to a decreasing trend. ***p < 0.001, **p < 0.01, *p < 0.05, ns—not significant. Perindopril demonstrates the most pronounced positive trend (τ = 0.762, p < 0.001), while Parnavel demonstrates the most pronounced negative trend (τ = −0.630, p < 0.001).

An analysis of search query trends for ACE inhibitors (Kendall’s correlation coefficient τ) showed that the strongest positive trend was recorded for perindopril (τ = 0.762 (95% CI 0.712–0.806), p < 0.001), indicating the most dynamic growth in interest in this drug (Figure 3). The strongest negative trend was identified for parnavel (τ = −0.630 (95% CI -0.707 to −0.536), p < 0.001), which may indicate a significant decline in interest in this drug. For captopril (τ = 0.539 (95% CI 0.450–0.616)), capoten (τ = 0.465 (95% CI 0.356–0.559)) and lisinopril (τ = 0.357 (95% CI 0.232–0.476)) also showed consistent positive trends, p < 0.001.

The analysis revealed a gradual decline in the relative importance of enalapril. Despite the continued high absolute figures, the share of search queries for individual enalapril brands within the overall information flow showed a trend toward a gradual decline by 2024–2025. In particular, a negative trend was identified for the drug “Enap” (τ = −0.171 (95% CI -0.306 to −0.0235), p = 0.022). At the same time, a positive trend was observed for the INN of enalapril (τ = 0.271 (95% CI 0.140–0.400), p < 0.001), which may reflect a shift among users from searching for specific brands to searching by active ingredient (Figure 3).

### 3.3 Variability of search interest

Time series analysis (Table 3) revealed significant differences in the variability of search interest among the drugs. The lowest coefficient of variation (CV), indicating the most stable and uniform interest, was recorded for fosinopril (CV = 17.1%), enalapril (19.1%), and lisinopril (21.9%). Conversely, the greatest variability was observed for perindopril (CV = 46.9%) and ramipril (39.3%), which is attributed to pronounced growth trends and/or sensitivity to external informational influences. The extremely high coefficient of variation for parnavel (CV = 67.7%) reflects a sharp decline in interest in this drug after 2020.

**TABLE 3:** Descriptive statistics for search queries on major ACE inhibitors (November 2018– October 2025, n = 84 months).

Drug	Average monthly search volume (M ± SD)	Median [Q1; Q3]	Coefficient of variation, %	Trend (Kendall’s τ)
Captopril	194,187 ± 48,580	192,148 [162,278; 228,055]	25.0	+0.539***
Capoten	172,507 ± 37,895	171,256 [146,405; 197,750]	22.0	+0.465***
Enalapril	164,509 ± 31,437	162,015 [138,029; 186,765]	19.1	+0.271***
Lisinopril	131,861 ± 28,840	131,210 [112,082; 153,774]	21.9	+0.357***
Perindopril	152,944 ± 71,670	150,413 [89,733; 202,304]	46.9	+0.762***
Ramipril	30,288 ± 11,914	28,006 [25,070; 34,069]	39.3	+0.354***
Fosinopril	7,431 ± 1,270	7,624 [6,596; 8,337]	17.1	+0.007 (ns)
Parnavel	3,637 ± 2,461	2,507 [1,505; 6,132]	67.7	−0.630***

The asymmetry and kurtosis indices indicate the nature of the distribution: for most drugs (captopril, enalapril, lisinopril, perindopril), the distribution is nearly symmetric (asymmetry ranging from −0.03–0.41), whereas for Ramipril (4.68), Amprilan (6.17), and Hartil (3.92), pronounced positive asymmetry is observed, caused by isolated peaks (likely associated with data points). High excess values for Amprilan (47.5), Ramipril (33.7), and Hartil (23.9) confirm the presence of “heavy tails” in the distribution—rare but extremely high values.

Thus, the analysis conducted may indirectly reflect the structure of media interest potentially linked to the clinical practice of ACE inhibitor use in Russia, where captopril and enalapril retain their status as the most sought-after drugs in the media landscape, which is explained by their long-standing presence on the market and wide availability. At the same time, lisinopril and perindopril demonstrate steady growth, which may indicate interest in these drugs in accordance with the principles of rational pharmacotherapy (the shift toward once-daily medications).

### 3.4 Comparative analysis of seasonal fluctuations in search activity

**FIGURE 4:**
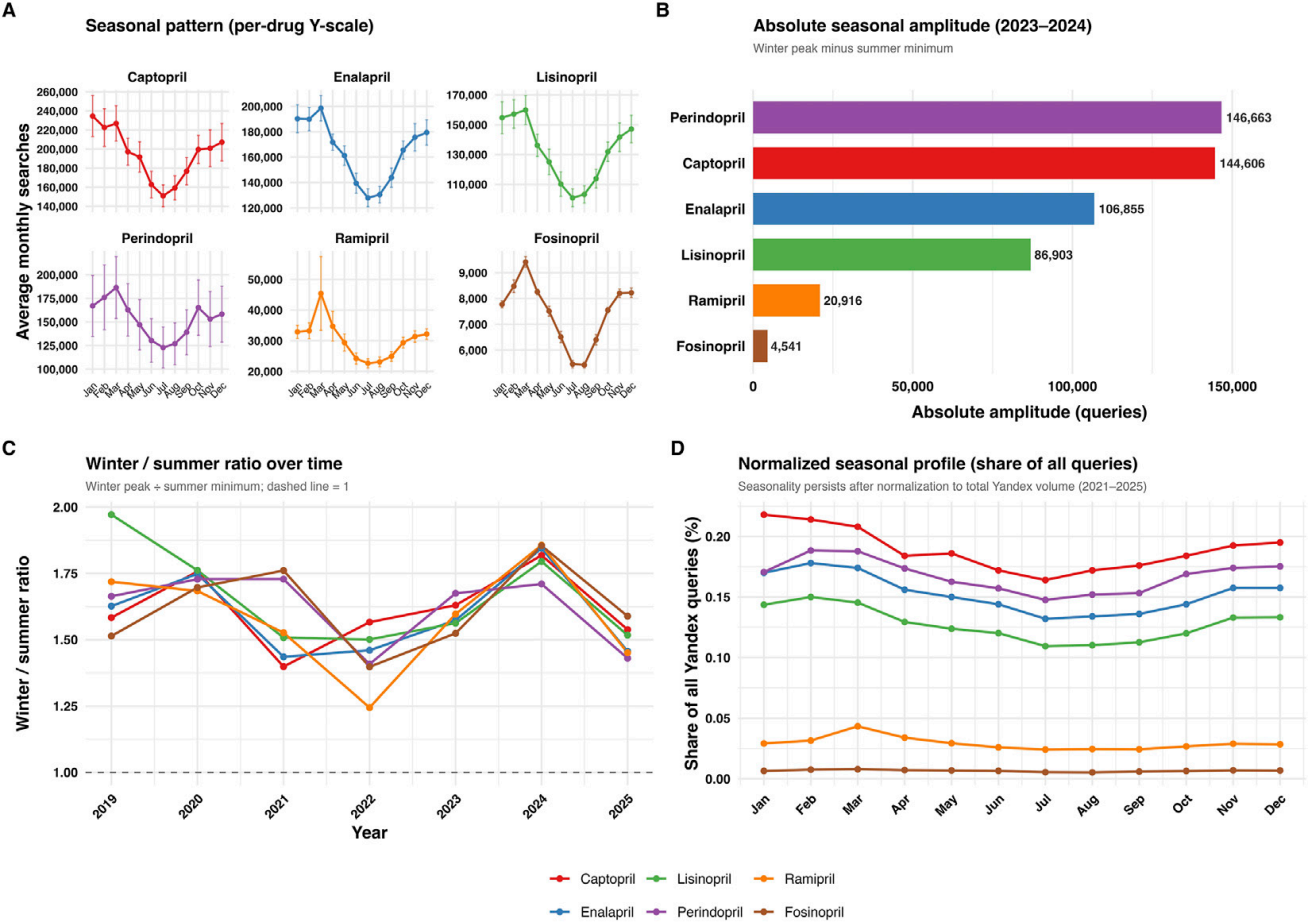
Seasonal analysis of search queries for ACE inhibitors. **(A)** Average monthly number of search queries for six ACE inhibitors (captopril, enalapril, lisinopril, perindopril, ramipril, fosinopril) for the period from November 2018 to October 2025. A separate vertical axis scale is used for each drug to clearly demonstrate seasonal fluctuations. The error bars represent the standard error of the mean (SE). **(B)** A bar chart illustrating the absolute seasonal amplitude (the difference between the winter peak and the summer minimum) for all six ACE inhibitors based on data from 2023 to 2024. **(C)** A line graph showing the winter-to-summer ratio for all six drugs over the 2019–2025 period. The dotted line at level 1 indicates that winter and summer levels are equal. Values above 1 indicate that the winter peak exceeds the summer low. **(D)** A line graph of the normalized seasonal profile—the share of search queries for each drug out of the total number of all Yandex queries for the corresponding month (%), averaged over the 2021–2025 period. This indicator eliminates the overall growth and seasonality of internet activity, since the total volume of Yandex queries is in the denominator. The persistence of seasonal fluctuations in the normalized series confirms that seasonality is specific to information interest in ACE inhibitors and is not reducible to the seasonality of general internet traffic.

**FIGURE 5:**
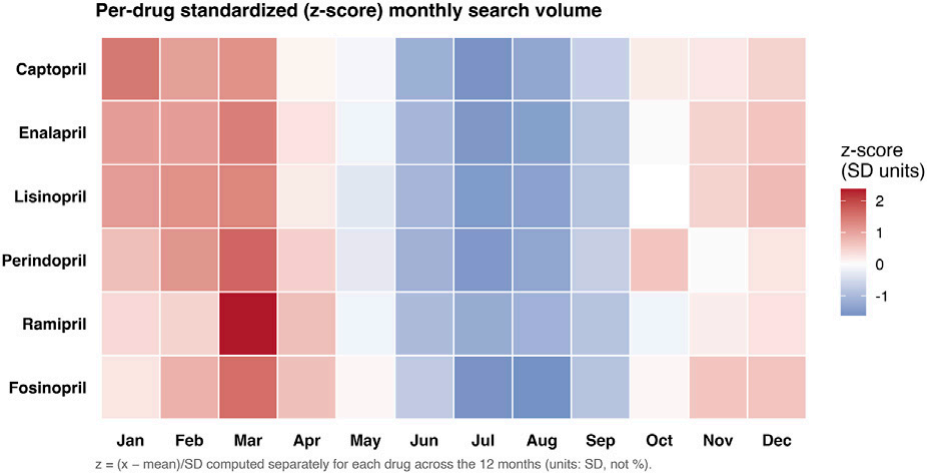
Heat map of normalized (z-score) average monthly search activity for ACE inhibitors. A heat map showing the normalized (z-score) average monthly search query values for each of the six drugs. Red indicates above-average query levels for a given drug, while blue indicates below-average levels. The z-score is calculated separately for each drug (z = (x − mean)/SD over 12 months). Units are standard deviations (range approximately from −1.6 to +2.4). Normalization is used solely to compare the seasonal profiles of drugs with different search volumes and does not represent normalization relative to overall internet activity.

**FIGURE 6:**
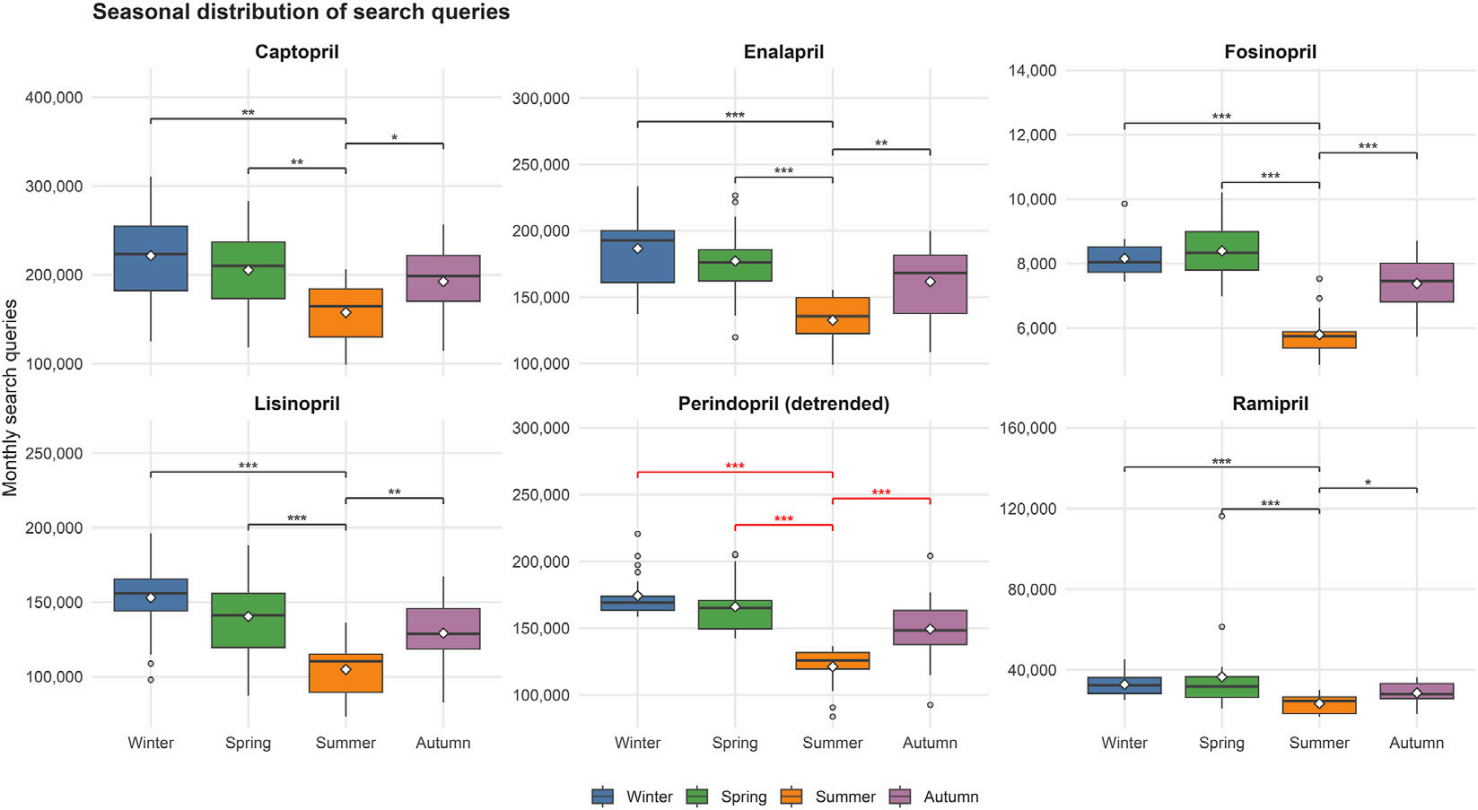
Seasonal distribution of search queries for key ACE inhibitors. Box plots show the distribution of monthly search volumes by season for captopril, enalapril, lisinopril, perindopril, ramipril, and fosinopril (November 2018–October 2025). Statistically significant differences between seasons are indicated by brackets with symbols: ***p < 0.001, **p < 0.01, *p < 0.05 (Wilcoxon test with Bonferroni correction for six pairwise comparisons). The most pronounced seasonal dynamics (winter–summer and spring–summer differences, p < 0.001) were identified for enalapril, lisinopril, and fosinopril. For ramipril and captopril, the differences were also significant (p < 0.01–0.001). The panel for perindopril is based on detrended data (STL, with a return to the mean): in the raw data, its seasonality is masked by a pronounced upward trend (τ = 0.762), whereas after detrending it becomes highly significant (red brackets; winter–summer, spring–summer, and summer–fall: p < 0.001; ε^2^ = 0.61–0.83). On the boxplots, the horizontal line is the median, the rectangle’s boundaries are Q1 and Q3 (interquartile range), the “whiskers” are 1.5×IQR, and individual points are outliers; the white diamond denotes the arithmetic mean.

An analysis of time series revealed a pronounced cyclical component in the dynamics of search queries related to ACE inhibitors. The universality of the identified pattern is confirmed by its synchronous presence for most of the studied INNs and their commercial brands. The peak phase of information activity consistently falls between November and March, with maximum absolute values in December–February. The remission phase, characterized by a minimum of queries, is observed during the summer months (June–August), with a nadir in July. Thus, the greatest public interest in ACE inhibitors is observed during the fall and winter months (Figures 4–6).

Statistically significant seasonal variations (p < 0.05) were observed for most of the 21 drugs analyzed. The exception was parnavel (p > 0.05). For perindopril, seasonal variations did not reach statistical significance in the analysis of the original time series (χ^2^ = 5.52, p > 0.05); however, after removing the upward trend (τ = 0.762), seasonality became highly significant (χ^2^ = 68.9, p < 0.001), indicating a masking effect of the trend. The detrended panel for perindopril in Figure 6 (red brackets) clearly demonstrates this effect: after removing the trend, the seasonal differences between winter–summer, spring–summer, and summer–fall become highly significant (p < 0.001). For parnavel, seasonal differences are already absent in the original series (χ^2^ = 5.8, p > 0.05), indicating the absence of pronounced seasonality; interpretation for this drug is further limited by a sharp decline in interest after 2020.

A *post hoc* analysis (pairwise Wilcoxon rank-sum (Mann–Whitney U) tests with Bonferroni correction) demonstrated a consistent pattern for all drugs with confirmed seasonality: the greatest differences were observed between the summer and winter periods (p < 0.001 for most drugs), as well as between summer and spring (p < 0.001) (Figure 6).

The most significant absolute range of seasonal fluctuations is characteristic of captopril, capoten, perindopril, and enalapril—drugs that lead in total search volume.

To quantitatively assess seasonality for the 2021–2025 period (excluding the anomalous pandemic year of 2020), two complementary indicators were calculated: the ratio of the number of queries in winter to summer (winter/summer) and the relative seasonal decline (percentage decrease from the winter peak to the summer trough). The winter/summer ratio exceeded 1.35 for all drugs, indicating significantly higher search activity during the winter months. The highest ratio values were observed for lisinopril (1.45; 95% CI: 1.37–1.54), captopril (1.43; 95% CI: 1.32–1.54), enalapril (1.41; 95% CI: 1.35–1.48), fosinopril (1.39; 95% CI: 1.29–1.50), and ramipril (1.38; 95% CI: 1.27–1.51); the lowest was for perindopril (1.36; 95% CI: 1.09–1.67).

The relative seasonal decline was as follows: lisinopril — 31.2% (95% CI: 27.0%–35.1%), captopril—30.2% (95% CI: 24.1%–35.5%), enalapril — 29.2% (95% CI: 25.7%–32.8%), fosinopril — 27.9% (95% CI: 22.0%–33.2%), ramipril — 27.6% (95% CI: 21.2%–33.6%), and perindopril — 26.3% (95% CI: 7.7%–40.8%). The wider confidence interval for perindopril reflects the masking effect of a pronounced upward trend (τ = 0.762); after removing this trend, the seasonal effect becomes highly significant (ε^2^ = 0.83).

The magnitude of seasonal variation, as measured by the epsilon-squared (ε^2^) statistic, ranged from moderate to strong: enalapril—ε^2^ = 0.664, lisinopril—ε^2^ = 0.655, fosinopril—ε^2^ = 0.580, captopril—ε^2^ = 0.574, ramipril—ε^2^ = 0.534 (strong effect), and perindopril—ε^2^ = 0.121 (moderate effect). The lower effect for perindopril is explained by the masking effect of a pronounced upward trend; after detrending, ε^2^ increases to 0.83, confirming the presence of strong seasonality. These data indicate that the identified seasonal differences are statistically significant (p < 0.001 for all drugs after detrending).

The normalized seasonal amplitude (the proportion of queries out of the total number of queries on Yandex for the month) helps eliminate the impact of the overall increase in internet activity. The highest normalized amplitude was recorded for captopril, enalapril, and lisinopril, confirming their key role in seasonal trends. Furthermore, an analysis of the dynamics of the share of queries reveals a long-term trend toward an increase in the relative importance of the ACE inhibitors topic during the winter period. For example, the share of queries related to perindopril during the winter months of 2023–2024 consistently exceeded the peak values of 2018–2019.

To verify whether the identified seasonal fluctuations were merely a reflection of the well-known seasonal decline in overall internet activity (mid-December and July–August), we additionally conducted a seasonal analysis on a normalized series—the share of queries for each drug out of the total number of all queries in the Yandex search engine for the corresponding month. By design, this metric eliminates both long-term growth and the seasonality of aggregate search traffic. After such normalization, statistically significant seasonality (Kruskal–Wallis test, p < 0.001) persisted for five of the six INNs (enalapril, lisinopril, fosinopril, ramipril, captopril). For perindopril, as with the absolute data, seasonality was masked by a pronounced upward trend (p = 0.38). The effect size decreased as expected (ε^2^ from 0.32 to 0.54 versus 0.53–0.66 for absolute values), and the relative seasonal decline in market share was 16.5%–20.8% (versus 27.6%–31.2% for absolute values). Thus, part of the observed fluctuations is indeed explained by the general seasonality of internet activity; however, a significant drug-specific seasonal signal persists and cannot be reduced to the dynamics of aggregate internet traffic.

To assess the potential impact of external events on search activity, we conducted an additional analysis comparing the number of search queries during the first wave of the COVID-19 pandemic (February–April 2020) with the same period in 2019. The Wilcoxon test did not reveal any statistically significant differences for any of the six INNs (p > 0.05 for captopril, enalapril, lisinopril, perindopril, ramipril, and fosinopril). Thus, the COVID-19 pandemic did not have a detectable impact on the overall patterns of search queries for ACE inhibitors within the limits of this analysis.

Thus, the seasonality of interest in ACE inhibitors is a statistically significant and reproducible digital pattern that reflects cyclical changes in the population’s information-seeking behavior. The consistency of this pattern across most ACE inhibitors suggests the presence of common external factors, which could potentially include seasonal fluctuations in blood pressure, as well as changes in the frequency of medical consultations and levels of health concern during the colder months of the year; however, further research incorporating clinical and epidemiological data is needed to establish precise cause-and-effect relationships.

### 3.5 Semantic analysis

To qualitatively interpret the structure of search interest, a content analysis was conducted on the complete list of relevant search queries provided by the Yandex. Wordstat tool. The analysis was based on data from November 2025.

#### 3.5.1 Captopril: dosage, safety, and emergency use

Thematic modeling (LDA) of captopril was performed for five themes. Theme 1 reflects interest in dosage and analogues (β = 0.061 for “moxonidine,” β = 0.050 for “dose,” β = 0.037 for “analogue”). Theme 2 characterizes use for high blood pressure (β = 0.153 for “high,” β = 0.104 for “what blood pressure,” β = 0.102 for “take”). Theme 3 relates to dosage and duration of action (β = 0.156 for “how much,” β = 0.108 for “take,” β = 0.035 for “works”). Theme 4 includes practical information: instructions (β = 0.160), price (β = 0.089), reviews (β = 0.089), analogues (β = 0.058), and a reference to sublingual administration (“under the tongue,” β = 0.026). Theme 5 is characterized by a comparison with the original drug Capoten (β = 0.055) and questions about the method of administration (“dissolve” β = 0.015, “swallow” β = 0.012).

K-means clustering confirmed these patterns and additionally identified a unique cluster for safety and toxicity, including the words “burn,” “poisoning,” “suicide,” and “test.” A separate cluster is associated with sublingual administration (“sublingually”) and the manufacturer Velfarm.

K-medoids clustering based on the Levenshtein distance (k = 6) yielded a silhouette coefficient of 0.095. The cluster with the medoid “captopril for high blood pressure” (76 queries) reflects the use of the drug for hypertension.

**FIGURE 7:**
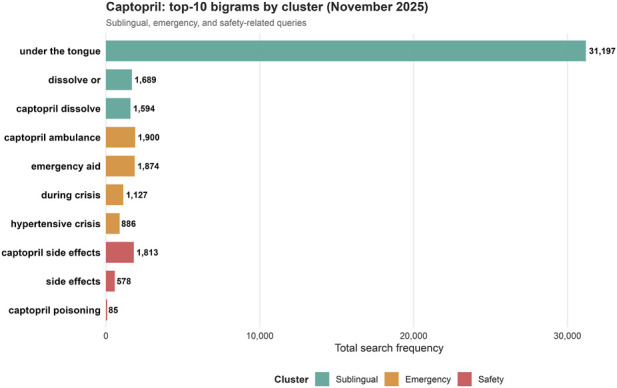
Top 10 bigram clusters for captopril.

Bigram analysis identified key patterns (Figure 7). The “Sublingual administration” cluster comprised 35,584 queries, of which 31,197 corresponded to the bigram “under the tongue.” The “Emergency care” cluster comprised 6,839 queries, including “captopril ambulance” (1,900), “ambulance” (1,874), “during a crisis” (1,127), and “hypertensive crisis” (886). The “Comparison” cluster comprised 42,117 queries (“or captopril” — 11,461, “which is better” — 7,022). The “Manufacturers” cluster comprised 33,820 queries (“captopril Velfarm” — 16,273). The “Safety” cluster comprised 2,701 queries (“captopril side effects” — 1,813, “captopril poisoning” — 85).

A quantitative analysis showed that 27.34% of queries were for instructions, 21.14% for dosage, and 12.06% for reviews. Targeted keyword analysis identified 24 unique emergency queries (1.20% of all unique queries, 0.53% of the total number of searches), dominated by the patterns “ambulance” (53% of emergency queries) and “crisis” (41.5%). The most frequent queries were: “captopril ambulance” (1,066), “captopril for a crisis” (994), and “captopril for a hypertensive crisis” (835). The share of requests regarding sublingual administration was 3.13%, which is a unique characteristic of captopril among all the analyzed drugs.

Thus, captopril is characterized by a unique combination of queries regarding dosage, sublingual administration, safety (toxicity, overdose), and emergency use, which distinguishes it from other ACE inhibitors.

#### 3.5.2 Enalapril: diversity of manufacturers and first-line therapy

Thematic modeling (LDA) of enalapril was performed for five themes. Theme 1 reflects use for high blood pressure (β = 0.170 for “what blood pressure,” β = 0.169 for “take,” β = 0.164 for “blood pressure”). Theme 2 is associated with the manufacturer Reneval (β = 0.059), side effects (β = 0.023), and combination with amlodipine (β = 0.020). Theme 3 includes instructions (β = 0.213), price (β = 0.149), reviews (β = 0.131), alternatives (β = 0.088), and comparison with perindopril (β = 0.002). Theme 4 is characterized by simple explanations (“simple” β = 0.082, “words” β = 0.045), the manufacturer Hexal (β = 0.078), and a comparison with losartan (β = 0.007). Theme 5 is associated with dosage (“how much” β = 0.085, “dosage” β = 0.049) and prescription format (“prescription” β = 0.018, “Latin” β = 0.011).

K-means clustering identified multiple clusters associated with manufacturers (Hexal, Reneval, Hemofarm, Pranapharm, Stada).

K-medoids clustering (k = 6) yielded a silhouette coefficient of 0.151 for enalapril. The most representative cluster associated with manufacturers had the medoid “enalapril hexal price” and included 77 unique search queries, confirming high interest in the specific Hexal brand.

Bigram analysis showed that the “Manufacturers” cluster accounted for 66,355 queries, which is one of the highest values among all medications. Key bigrams: “enalapril hexal” (43,645), “enalapril reneval” (10,809). The “Comparison” cluster accounted for 27,797 mentions (“enalapril analogues” — 8,550, “which is better” — 3,386). The “Fixed Combinations” cluster accounted for 5,624 queries (“enalapril amlodipine” — 2,553, “enalapril indapamide” — 1,926). The “Emergency Care” cluster was minimal—339 queries (“during a crisis”—183, “hypertensive crisis”—156).

A quantitative analysis showed that 33.96% of queries were about instructions, 19.96% about dosage, and 17.29% about reviews. The share of queries about manufacturers was 6.74%—the highest among all drugs, reflecting users’ active interest in generics (Hexal, Reneval, Hemofarm, Pranapharm, Stada). The share of queries about fixed-dose combinations was small (0.80%). Emergency queries accounted for 0.04%.

Enalapril occupies an intermediate position: it is associated with routine therapy but lags behind perindopril in the frequency of queries regarding combination therapy, while demonstrating the greatest diversity of interest in specific manufacturers.

#### 3.5.3 Lisinopril: combination therapy and high interest in instructions

Thematic modeling (LDA) of lisinopril was performed for 5 themes. Theme 1 reflects use for high blood pressure (β = 0.156 for “take,” β = 0.155 for “at what (blood pressure level)”) and comparison with enalapril (β = 0.024). Theme 2 includes the instructions (β = 0.456) and usage (β = 0.200). Theme 3 is characterized by combination with amlodipine (β = 0.028), indapamide (β = 0.013), and the manufacturer Vertex (β = 0.021). Theme 4 is associated with price (β = 0.375), simple explanations (“simple” β = 0.086, “words” β = 0.046), and comparison with the original brand Diroton (β = 0.004). Theme 5 includes reviews (β = 0.331), generics (β = 0.250), the manufacturer Teva (β = 0.028), and comparison with losartan (β = 0.008).

K-means clustering confirmed the significance of the combination with amlodipine, which appears in several clusters, and also identified queries comparing it with bisoprolol, telmisartan, and Concor.

K-medoids clustering (k = 6) yielded a silhouette coefficient of 0.172 for lisinopril. The cluster with the medoid “amlodipine lisinopril” (86 unique search queries) confirms the significance of combination therapy, which is consistent with the data from bigram analysis (7,165 queries for the bigram “amlodipine lisinopril”). Bigram analysis showed that the “Comparison” cluster comprised 47,363 queries (“lisinopril analogues” — 19,922, “which is better” — 9,949). The “Fixed Combinations” cluster comprised 13,638 mentions, of which “amlodipine lisinopril” accounted for 7,165 and “lisinopril indapamide” for 3,110. The “Manufacturers” cluster accounted for 23,704 queries (“lisinopril Teva” – 11,500, “lisinopril Vertex” – 8,244).

Quantitative analysis showed that the share of queries regarding instructions was 37.34%—the highest figure among all drugs, reflecting an active search for information on proper use. The share of queries regarding dosage was 20.87%, and regarding reviews—19.72% (also the highest figure). The share of queries regarding fixed-dose combinations (primarily with amlodipine) was 1.76%, and those regarding manufacturers was 3.12%. There were no emergency queries.

Lisinopril is characterized by a combination of queries regarding combination therapy (particularly with amlodipine), comparisons with other antihypertensive drugs (bisoprolol, telmisartan, concor), and an active search for simple explanations, as well as a high level of interest in the instructions and reviews.

#### 3.5.4 Perindopril: fixed-dose combinations as the dominant theme

Thematic modeling (LDA) of perindopril was performed for 5 themes. Theme 1 includes simple explanations (“simple” β = 0.294, “words” β = 0.226), information for patients (“adults” β = 0.079, “prescribed” β = 0.042), and side effects (β = 0.028). Theme 2 reflects instructions (β = 0.246), price (β = 0.220), and reviews (β = 0.160). Theme 3 is characterized by generics (β = 0.313) and fixed-dose combinations: indapamide (β = 0.120), amlodipine (β = 0.073), “plus” (β = 0.051). Theme 4 is associated with usage (β = 0.148) and manufacturers (Teva β = 0.104, Alium β = 0.010). Theme 5 includes comparisons with bisoprolol (β = 0.008) and Perineva (β = 0.005).

K-means clustering confirmed the dominance of fixed-dose combinations: the clusters include “indapamide,” “amlodipine,” “noliprel,” and “plus.” A separate cluster is associated with the side effect “cough.”

K-medoids clustering (k = 6) revealed a silhouette coefficient of 0.200 for perindopril—the highest among all drugs. The cluster with the medoid “perindopril indapamide price” (70 queries) reflects interest in the combination with indapamide.

**FIGURE 8:**
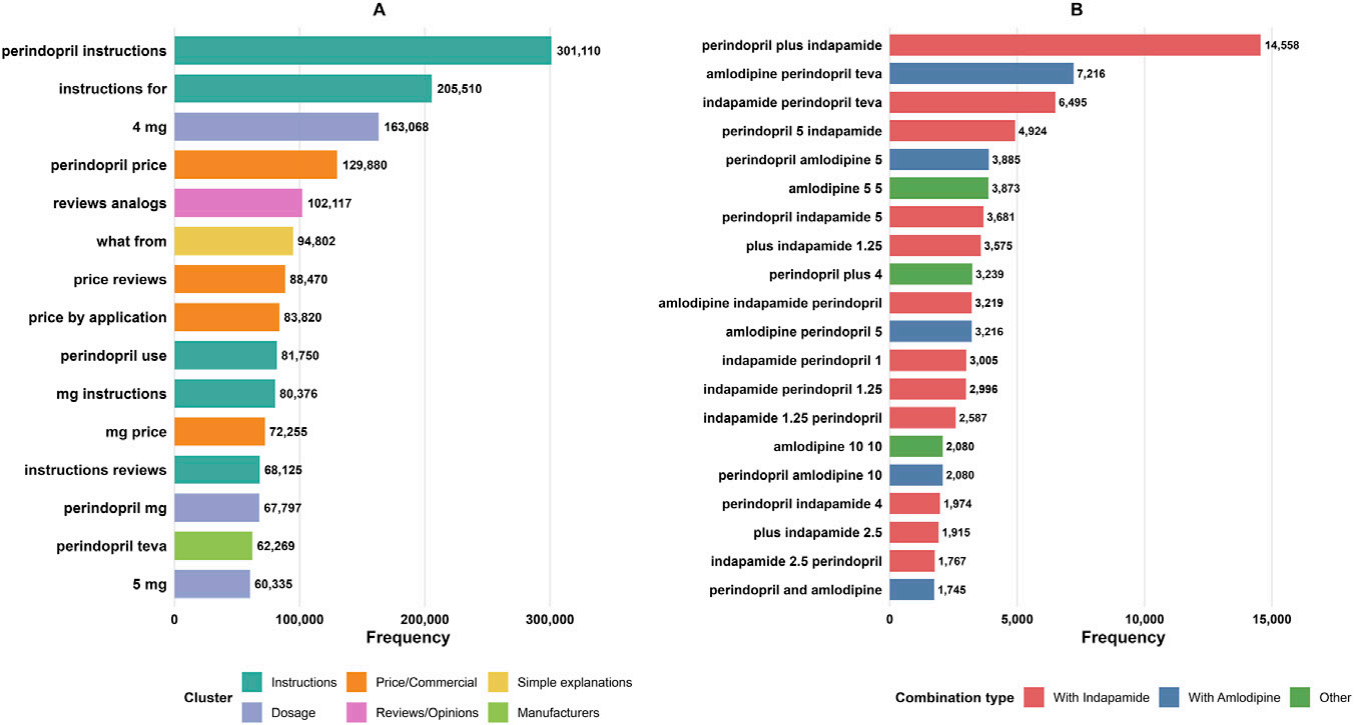
Semantic analysis of search queries for perindopril in November 2025. **(A)** — Top 15 bigrams. **(B)** — Top 20 trigrams of fixed combinations.

Bigram analysis showed the absolute dominance of the “Fixed Combinations” cluster—155,698 queries, which is 11 times higher than the figure for lisinopril (13,638) and 28 times higher than that for enalapril (5,624). Key bigrams: “perindopril plus” (42,182), “perindopril indapamide” (42,060), “perindopril amlodipine” (27,168), “amlodipine perindopril” (23,059), “indapamide perindopril” (21,229). The “Manufacturers” cluster accounted for 94,720 queries (“perindopril Teva” — 62,269, “perindopril Alium” — 6,514). The “Comparison” cluster accounted for 82,808 searches (“perindopril analogues” — 31,039, “which is better” — 11,584) (Figure 8).

The trigram analysis further detailed the structure of queries regarding fixed combinations. The most frequent trigrams were: “perindopril plus indapamide” (14,558), “amlodipine perindopril Teva” (7,216), “indapamide perindopril Teva” (6,495), “perindopril 5 (mg) indapamide” (4,924), “perindopril amlodipine 5 (mg)” (3,885), “amlodipine indapamide perindopril” (3,219). These data indicate that users are actively seeking information not only about fixed-dose combinations but also about specific dosages and manufacturers of combination drugs.

A quantitative analysis showed that the share of queries regarding instructions was 33.81%, regarding dosage—22.86%, and regarding reviews—18.22%. The share of queries about fixed-dose combinations was 10.81%—the absolute maximum among all drugs, reflecting the active use of perindopril in combination therapy. The most frequent queries in this cluster were: “perindopril indapamide” (42,060), “perindopril amlodipine” (27,168), “amlodipine perindopril” (23,059), “indapamide perindopril” (21,229). The share of queries about manufacturers was 4.77% (Teva, KRKA, Izvarino, Prana). There were no emergency queries at all.

Perindopril exhibits the most complex semantic structure, in which fixed-dose combinations with indapamide and amlodipine play a central role, as do active comparisons of the original drug Prestarium with generics (Perineva, Parnavel) and choices between manufacturers (Teva, KRKA, Izvarino, Prana).

### 3.6 Summary analysis of semantic patterns

A comparison of the results from all five methods revealed clear differences between the drugs (Table 2).

Captopril is the only drug most frequently associated with emergency use (0.53%) and sublingual administration (3.13%). Enalapril shows the greatest diversity of interest in specific manufacturers (6.74%, 66,355 mentions in the corpus). Lisinopril is characterized by high interest in instructions (37.34%) and reviews (19.72%). Perindopril absolutely dominates in terms of the frequency of queries regarding fixed-dose combinations (10.81% of all queries, 155,698 queries in bigrams), which is 11 times higher than that of lisinopril and 28 times higher than that of enalapril, reflecting its active use in combination therapy.

## 4 Discussion

This study represents a comprehensive longitudinal study of digital pharmacoepidemiology of ACE inhibitors in Russia. The results demonstrate persistent differences in the frequency, seasonality, and semantic structure of queries related to various representatives of the class, allowing search activity to be considered a potential source of additional information about patient engagement with pharmacotherapy.

One of the key observations is the marked variation in interest in individual medications. In particular, the higher frequency of queries related to captopril, combined with their characteristic semantic structure, may indicate specific patterns of information-seeking behavior regarding this medication. Given its pharmacokinetic characteristics (rapid onset and short duration of action) (Al-Furaih et al., 1991; Marte et al., 2024), it can be assumed that such search patterns may be linked to interest in emergency use. This digital pattern may reflect possible gaps in the population’s health literacy and in the “doctor-patient” communication system: a short-acting prescription drug is perceived as an over-the-counter “emergency aid in pill form,” though further research is needed to confirm this. It is also possible that the high number of queries on this topic indirectly indicates a shift in the treatment of hypertension from routine management aimed at controlling blood pressure within target ranges to crisis management of uncontrolled hypertension. Captopril is indeed an important drug used to treat hypertensive crises (Al-Furaih et al., 1991; Kaya et al., 2016; Mirdamadi et al., 2022; Diaz-Arocutipa, 2026), but the key question remains whether patients are receiving basic antihypertensive therapy or whether treatment is provided only on an emergency basis when blood pressure readings are very high. At the same time, it should be emphasized that search query data do not allow for a reliable determination of actual behavioral patterns, and the identified associations should be interpreted with caution.

Meanwhile, the observed growing interest in perindopril, particularly in its fixed-dose combinations, may hypothetically reflect a positive trend toward the gradual adoption of modern cardiology principles focused on managing overall cardiovascular risk (Thomson and Greenacre, 2007; Syed, 2022; Dat et al., 2024), however, independent data on actual prescribing practices are needed to confirm this hypothesis. The active discussion “Prestarium or Perineva” may serve as an example of how digital footprints potentially reflect patients’ decision-making processes at the intersection of brand trust, cost, and expected efficacy; however, this interpretation requires caution, as search queries are not direct evidence of actual behavior.

A second important finding from the current study relates to the seasonality of search interest in ACE inhibitors. The statistically confirmed universal seasonality of search queries for all ACE inhibitors, with a pronounced peak during the winter months, represents a potential digital indicator of seasonal fluctuations that could hypothetically be linked to blood pressure control at the population level. This phenomenon is fully consistent with known pathophysiological mechanisms (Rosenthal, 2004; Lewington et al., 2012; Fares, 2013; Stergiou et al., 2020). It is important to emphasize that search queries reflect informational interest rather than actual blood pressure monitoring. The significant scale of the observed fluctuations suggests that the current model of clinical follow-up, based on the principle of long-term monitoring, may not sufficiently account for this predictable cyclicality in information-seeking behavior. It remains unclear what accounts for the high public search activity for ACE inhibitors during the fall and winter: the emergence of new patients with newly diagnosed hypertension, the need for treatment adjustments in patients with an established diagnosis, or other factors. Answering this question requires further research incorporating clinical data. The results preliminarily indicate the potential value of developing proactive models; however, such models require further validation using independent data.

Thus, the seasonality of inquiries may be of potential clinical interest as a possible indicator for optimizing healthcare resources and improving the effectiveness of chronic disease management; however, this requires confirmation in studies that include data on the actual burden on the healthcare system.

Another observation from the current analysis could be tentatively termed the “paradox of digital adherence” (given the limitations of interpreting search data). On the one hand, the enormous volume of queries such as “instructions,” “how to take,” and “is it possible to … ” could hypothetically indicate patients’ high motivation to follow guidelines and their active involvement in the treatment process. On the other hand, the very fact that these fundamental questions are directed at a search engine rather than the treating physician may indicate a systemic lack of effective clinical communication and follow-up care. However, both of these interpretations are hypothetical, as search queries do not allow for a direct assessment of either patient motivation or actual communication with physicians.

The phenomenon of “crowdsourced pharmacovigilance,” manifested in widespread searches for “doctor reviews” and “alternatives,” may indicate that patients have access to an alternative, unreliable channel for making treatment decisions; however, its reliability and impact on actual decisions remain unknown. It is unclear what drives the public’s interest in searching for alternatives and why they do not consult their treating physician about this. The design of the current study does not allow us to determine whether these search efforts are aimed at improving the public’s health literacy or at forming an opinion alternative to that of the treating physician.

Previous studies have demonstrated the importance of patient health literacy. For example, a significant association was shown between health literacy and adherence to medication regimens in patients with ischemic heart disease (Zheng et al., 2020). Previous studies have shown that, despite high levels of concern regarding the adverse effects of ACE inhibitors, patients’ knowledge of these potential side effects was insufficient (Khoubaeva et al., 2012). Sixty-four patients (24.7%) reported the presence of at least one side effect of ACE inhibitors, only 94 patients (36.3%) had previously received any information from healthcare providers about at least one side effect of ACE inhibitors, and only 42% reported being aware of any side effects (Jankovic et al., 2019). Patients also frequently fail to adhere to treatment instructions (Engel et al., 2012). These findings from other studies confirm the relevance of the issues, but they do not replace the validation of our conclusions using independent data.

Despite the new and interesting data obtained, the current study has a number of limitations that must be taken into account when interpreting the results. First, search query data do not allow us to reliably determine whether users are patients, healthcare professionals, or other groups, which significantly limits the interpretation of the results as a reflection of actual patient behavior. Second, search activity is influenced not only by medical needs but also by marketing campaigns, drug availability in pharmacies, changes in prescription drug benefit programs, and media coverage. Third, the data reflect the behavior of the predominantly internet-active segment of the population, which may introduce a sampling bias. Fourth, the study identifies correlations and describes patterns but does not prove causal relationships between search queries and actual clinical decisions. Search queries do not necessarily translate into actual actions (e.g., taking a medication or changing therapy), which precludes the possibility of direct clinical conclusions. Furthermore, the semantic analysis was based on data from a single month. Although this month is representative of the onset of seasonal growth in search queries, the structure of queries may change over time, requiring confirmation across multiple time periods in future studies. It should be emphasized that this study was not cross-referenced with independent sources of data on actual medication use—such as prescription databases, pharmacy dispensing records, registries of subsidized medication provision, and prevalence rates of hypertension. Therefore, a correlation between search activity patterns and actual drug use cannot be established.

When interpreting the temporal trends, a number of potential confounding factors should be taken into account. The volume of search queries during the study period may have been influenced by: the COVID-19 pandemic and related changes in the healthcare system; fluctuations in the availability of certain medications and periods of drug shortages; changes in clinical guidelines and prescribing practices; import substitution policies, and the overall growth and seasonality of the population’s internet activity. Although a direct comparison with the period of the first wave of COVID-19 did not reveal any significant differences, and normalization against total search traffic confirmed the persistence of seasonality, it is impossible to completely rule out the influence of the listed factors based solely on search data.

On the other hand, the data obtained opens up several promising avenues for further research and practical implementation. The methodology of digital epidemiology holds great potential for objectively evaluating the effectiveness of government and medical initiatives. Monitoring the digital footprint in response to the launch of targeted information campaigns (e.g., on the risks of self-medication), changes in clinical guidelines, or lists of subsidized medications will allow for a quantitative assessment of their actual impact on information-seeking behavior and, indirectly, on patient awareness. In addition, in-depth semantic and network analysis can be applied to map entire ecosystems of queries related to cardiovascular diseases. This includes studying associative chains (e.g., from “high blood pressure” to “captopril,” and further to “heart attack” or “stroke”), which will allow us to model patients’ decision-making pathways and identify key points for timely informational interventions. Finally, the development and validation of artificial intelligence (AI)-based predictive models for analyzing time series of search queries represents a strategic direction. Such models could predict seasonal spikes in hypertensive decompensation, potential surges in medical care requests, or shortages of certain medications in pharmacy chains, enabling the healthcare system to transition from reactive to proactive and predictive resource management.

From a practical standpoint, the findings highlight the potential importance of taking patients’ information-seeking behavior into account when developing communication strategies and educational programs. In particular, differences in semantic patterns may indicate a potential need for more targeted patient education regarding medication usage guidelines, efficacy, and safety. However, any clinical or organizational conclusions based on such data require mandatory validation using independent sources of information.

Thus, the study results confirm the potential of search query analysis as an additional tool for studying information behavior in the field of pharmacotherapy. At the same time, to enhance the reliability and practical applicability of such approaches, their integration with clinical, pharmacoepidemiological, and sociological data is necessary.

Unlike previous infodemiological studies, which focused on general trends in disease development, this study presents a drug-specific, longitudinal, and semantically enriched analysis of the public’s information needs. The integration of temporal dynamics, seasonality, and multilevel semantic modeling offers a new perspective on digital traces as a potential source of information about therapeutic perceptions and the potential real-world use of medications; however, this interpretation requires validation.

## 5 Conclusion

This infodemiological study, a retrospective analysis of search queries over a 7-year period, confirms the high informational value of digital footprint data for population-based pharmacoepidemiology. The results revealed stable, statistically significant patterns in the population’s information behavior regarding ACE inhibitors in the Russian Federation.

Captopril has been found to dominate the information space, which may indicate its well-established role as a treatment for the acute management of hypertensive crises. A clear hierarchy and semantic specification of interest in drugs within this group have been identified, forming distinct conditional information patterns: from a pattern associated with crisis-related interest in captopril, through the archetype of accessible baseline therapy (enalapril) and the image of a modern component of combination therapy (lisinopril), to a complex, combinatorial perception of perindopril.

A universal seasonal trend has been statistically confirmed, with search activity peaking during the winter months (November–March). The relative seasonal decline in search queries during the summer ranged from 26.3% (perindopril) to 31.2% (lisinopril). After eliminating long-term trends, seasonal differences become highly significant for all key medications (p < 0.001). This phenomenon can be viewed as a potential (requiring validation) population-based digital indicator of cyclical changes in information interest potentially related to blood pressure control, and may indicate the advisability of developing and implementing proactive, seasonally adapted models of outpatient monitoring for patients with hypertension.

The high volume of inquiries regarding clarification of instructions, dosages, alternatives, and reviews may indicate that patients are actively seeking information on their own during the course of treatment. This observation potentially highlights the need to optimize communication channels between doctors and patients and to increase the availability of verified educational resources to ensure informed adherence to treatment.

Thus, the study results confirm the potential of search query analysis methodology as an additional tool for studying information-seeking behavior, which requires further validation in combination with clinical and epidemiological data. The findings may serve as a basis (requiring further validation) for developing targeted medical education interventions (Olumide et al., 2025), improving pharmacovigilance systems, and planning healthcare resources in light of the identified digital patterns.

## Data Availability

The original contributions presented in the study are included in the article/supplementary material, further inquiries can be directed to the corresponding author.
